# Structures of Gate Loop Variants of the AcrB Drug Efflux Pump Bound by Erythromycin Substrate

**DOI:** 10.1371/journal.pone.0159154

**Published:** 2016-07-12

**Authors:** Abdessamad Ababou, Vassilis Koronakis

**Affiliations:** Department of Pathology, University of Cambridge, Tennis Court Road, Cambridge, United Kingdom; Centre National de la Recherche Scientifique, Aix-Marseille Université, FRANCE

## Abstract

Gram-negative bacteria such as *E*. *coli* use tripartite efflux pumps such as AcrAB-TolC to expel antibiotics and noxious compounds. A key feature of the inner membrane transporter component, AcrB, is a short stretch of residues known as the gate/switch loop that divides the proximal and distal substrate binding pockets. Amino acid substitutions of the gate loop are known to decrease antibiotic resistance conferred by AcrB. Here we present two new AcrB gate loop variants, the first stripped of its bulky side chains, and a second in which the gate loop is removed entirely. By determining the crystal structures of the variant AcrB proteins in the presence and absence of erythromycin and assessing their ability to confer erythromycin tolerance, we demonstrate that the gate loop is important for AcrB export activity but is not required for erythromycin binding.

## Introduction

AcrAB-TolC is the major multidrug resistance efflux pump in *Escherichia coli*, where AcrB is the efflux transporter residing in the inner membrane of the bacterium, playing an important role in capturing and expelling a range of structurally diverse substrates [1–7]. AcrB is homotrimeric with the three monomers suggested to cycle between three conformational states known as loose/access, tight/binding, and open/extrusion during substrate export [8–10]. Crystallographic studies of AcrB have revealed that large substrates e.g. erythromycin bind the proximal pocket in the access/loose monomer (access binding pocket) while smaller substrates e.g., doxorubicin and minocycline bind the distal pocket in the binding/tight monomer (deep binding pocket) [8, 10].The proximal and distal sites are separated by a short “gate” loop (herein defined to consist of residues ^615^FGFAGR^620^) that has been suggested to regulate the progress of substrates through the export pathway [8, 10].

During efflux, large substrates such as erythromycin are proposed to undergo a sequential export process, first binding the proximal pocket, then passing the gate loop to the distal pocket, and subsequently being extruded out of the cell via TolC [8, 10]. Multiple reports have shown that mutation or deletion of residues in the gate loop negatively affect AcrB export activity [8, 10–14].

The gate loop is also known to be involved in substrate binding. For example, in previously reported crystal structures of rifampicin, erythromycin, and doxorubicin-bound AcrB, the central phenylalanine (Phe617) makes inter-molecular interactions with these antibiotics when bound in the proximal site [8, 10]. Removal of the phenylalanine side chain has been reported to significantly affect efflux, both alone and in the context of multiple amino acid substitutions [11, 12].

To further probe the importance of the gate loop in AcrB-mediated antibiotic resistance, we constructed two novel gate loop variants. The first, AcrB(AAA), contains three mutations (F615A-F617A-R620A) chosen to remove bulky side chains from the gate loop. The second, AcrB(ΔLoop), lacks the entire gate loop, due to substitution of residues 615–620 by a single glycine. We then used X-ray crystallography to solve the structures of each gate loop variant to assess their ability to bind the antibiotic substrate, erythromycin. Furthermore, we assessed the ability of the variants to complement AcrB-deficient strains.

## Methods

### Cloning, expression and purification of AcrB and gate loop variants

Plasmid pETHisAcrB [15] expresses C-terminally His-tagged AcrB and was used as template for Quikchange site-directed mutagenesis to produce two gate loop variants, F615A-F617A-R620A (AAA), and F615G-Δ(G616-R620) (ΔLoop). Successful production of variant plasmids was confirmed by DNA sequencing (Source Bioscience). To express wild type AcrB or gate loop variants, *E*. *coli* C43 cells bearing the appropriate plasmid were grown at 30°C in 2TY medium containing 50 mg/l carbenicillin to an OD600 of 0.6, and then induced with 0.5 mM IPTG for 4 h. Cells were harvested by centrifugation and the pellets resuspended in 20 mM Tris pH 8.0, 500 mM NaCl, 5% (v/v) glycerol, 2 mM MgCl_2_. Cells were broken by passage through a cell disruptor at 30,000 psi. After centrifugation at 10,000xg for 10 min at 4°C, the supernatant was subjected to ultracentrifugation at 140,000xg for 1 h. The membrane pellet was resuspended and solubilised in 10 mM potassium phosphate pH 8.0, 100 mM NaCl, 10 mM imidazole and 1.0% (w/v) n-dodecyl-β-D-maltoside (DDM) for 1 h at 4°C and centrifuged for 1 h at 145,000xg. The supernatant, containing the detergent-solubilised membrane fraction was applied to a Ni-NTA agarose column pre-equilibrated with buffer A (20 mM potassium phosphate pH 8.0, 100 mM NaCl, 10 mM imidazole, 0.02% (w/v) DDM). The column was washed with buffer B (20 mM potassium phosphate pH 8.0, 300 mM NaCl, 50 mM imidazole, 0.02% (w/v) DDM), and the protein eluted with buffer C (20 mM potassium phosphate pH 8.0, 100 mM NaCl, 300 mM imidazole, 0.02% (w/v) DDM). Proteins were buffer exchanged into 20 mM Tris pH 8.0, 100 mM NaCl, 0.03% (w/v) DDM, using an Amicon 100 kDa molecular weight cut-off concentrator (Millipore).

### Crystallization of AcrB and gate loop variants

Crystallization trials were conducted using commercially-available crystallization screens and were performed using sitting drop vapour diffusion methods in 96 well MRC plates employing Mosquito crystallisation robotics to set the drops. Crystallisation ‘hits’ were then manually optimized in 24 well plates. Wild type, AcrB(AAA), and AcrB(ΔLoop) crystals were grown by the hanging drop vapour diffusion method at 15°C, using protein concentrations of 29, 24, and 20 mg/ml, respectively. The drops were made by mixing 2 μl of protein solution with 2μl of crystallization reagent, and equilibrated against 1 ml of the crystallization reagent alone. Crystallization of AcrB used a crystallization reagent composed of 0.1 M MES pH 6.5, 0.2 M magnesium acetate, 10% (w/v) PEG 3350. Crystals of the AcrB and variants complexes with erythromycin were obtained by soaking apo-protein crystals in an identical buffer supplemented with 10 mM erythromycin and 1% (v/v) glycerol or ethylene glycol. The crystals were incubated at 15°C for up to 2 weeks, and then cryoprotected by stepwise addition of a cryoprotectant solution composed of 0.1 M MES pH 6.5, 0.2 M magnesium acetate, 10% (w/v) PEG 3350, 10 mM erythromycin supplemented with either 22% (v/v) glycerol or 25% (v/v) ethylene glycol prior to flash freezing inliquid nitrogen.

### X-ray data collection, phasing and refinement

X-ray diffraction data for screening and checking crystals quality were collected on beam line ID23-1 at the European Synchrotron Radiation Facility (Grenoble, France). X-ray diffraction data were collected at 100 K on beam line PXIII at Swiss Light Source (SLS) at the Paul Scherrer Institut (Villigen PSI, Switzerland), and on beam line I02, I03, and I04 at Diamond Light Source (Didcot, UK). X-ray data sets were indexed and integrated using iMosflm [16] and scaled using Scala or Aimless in the CCP4 suite [17]. The structures were solved by molecular replacement using either Phaser[18] or Molrep[19]. Structures of P21 crystal forms were solved using PDB file 2GIF [20] with the phasing search using separate monomers (A, B, and C), consisting of residues 2–1033, instead of the whole trimer as the search molecule. All Structures refinement were performed in two steps: First we used the recently reported refinement strategy combining the Rosetta sampling methodology and energy function with reciprocal-space X-ray refinement in Phenix [21], and then we continue the refinement using Phenix [22]. The structures were completed with iterative rounds of manual model-building with Coot [23] and refinement in Phenix. The presence of erythromycin in datasets corresponding to erythromycin-soaked crystals was confirmed using the weighted |*F*_*o*_|-|*F*_*c*_| difference map prior to its placement in the structure. Omit maps were made by removing the erythromycin from the final refined models and calculating a weighted difference map after a single round of refinement. Structural figures were prepared using PyMol (www.pymol.org). Refined structures of AcrB and the two gate loop variants have been deposited with the Protein Data Bank with accession codes 4ZIT, 4ZIV, 4ZIW. The corresponding erythromycin-bound structures have accession codes 4ZJL, 4ZJO, 4ZJQ.

### Antibiotic susceptibility

Single colonies of *acrAB*-knockout *E*. *coli* MC1061 [15] strains harbouring pET15b-based plasmids encoding histidine-tagged AcrB constructs (either wild type pETHisAcrB [15] or gate loop variants) under control of T7 Lac promoter, and a separate pACYC184-based plasmid encoding wild type AcrA (pACT7AcrA [15]), were grown overnight at 37°C in 2TY medium supplemented with 4μg/ml chloramphenicol and 25μg/ml carbenicillin. Overnight cultures were subsequently used to inoculate 10 ml fresh 2TY medium and grown at 37°C to an OD600 of 0.6–0.8. Cultures were incubated for a further 30 min in the presence of 0.5 mM IPTG to induce AcrAB expression before diluting into wells of a 96-well plate containing successive 2-fold serial dilutions of 2mM erythromycin. IPTG was maintained at 0.5 mM. Bacterial growth was quantified by OD600 measurements using a microplate reader and inhibition defined as having an OD600 less than 0.04 after growth overnight. Experiments were conducted in triplicate.

### Western blot of AcrB and variants

Cultures of MC1061*ΔacrAB* [15] carrying pACT7AcrA and either wild-type AcrB (pETHisAcrB [15]), variant AcrB, or an empty vector (pET15b) were grown under similar conditions to those used for MIC determination. After reaching an OD600 of 0.8, cultures were incubated for a further 30 min in the presence of 0.5 mM IPTG before harvesting by centrifugation. The bacterial cell pellet was resuspended in SDS-PAGE loading buffer separated by SDS-PAGE and blotted onto nitrocellulose membrane. AcrB and its variants were immunodetected with anti-AcrB rabbit primary antibody specifically raised for the detection of *E*. *coli* AcrB [15] (1 in 10^4^ dilution) and IR-labelled secondary antibodies (LI-COR, Biosciences) as recommended by the manufacturer. The blot was scanned using LI-COR Odyssey.

## Results and Discussion

### Structures of AcrB and its gate loop variants

**Fig 1:**
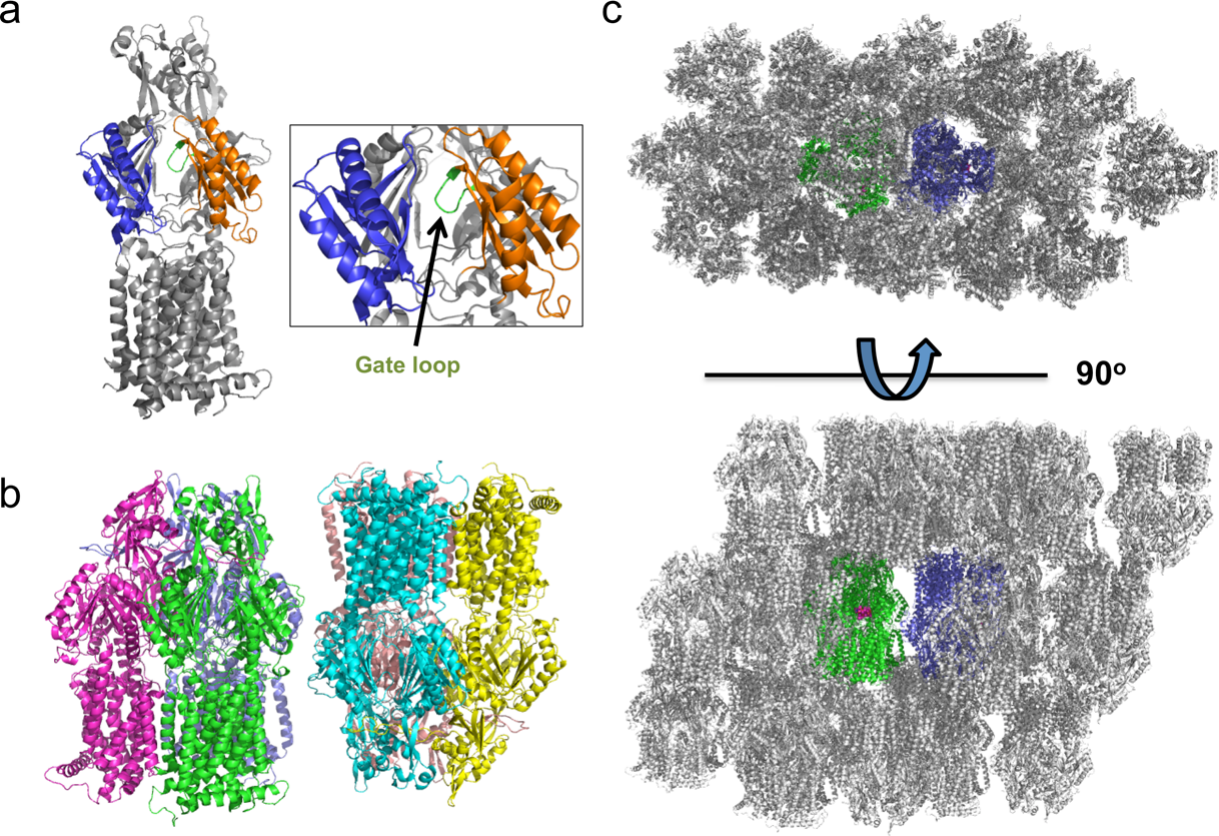
Structure of AcrB in space group P2_1_. (**a**) Location of the AcrB gate loop. Porter domains of the periplasmic domain are shown in *blue* and *orange*, the gate loop is shown in *green*. (**b**) The asymmetric unit of the P2_1_ crystal form of AcrB composed of two trimers. Each monomer is individually colored. (**c**) Crystal packing and binding site accessibility. Erythromycin is shown in magenta.

Wild type AcrB and both gate loop variants were crystallised in a novel crystal form at pH 6.5 (**Fig 1**). Crystals of AcrB belong to space group P2_1_ with two AcrB trimers (6 molecules) per asymmetric unit. Overall, the two trimers appear very similar to one another and can be superposed with an rmsd of 0.55 Å (for 3119 matched Cα positions). At least one crystal contact appears to result from the presence of an electron dense metal ion, most likely ascribable to a nickel ion carried over during affinity purification. The C-terminus is also unusually well-ordered in this crystal form, allowing the majority of the C-terminal residues to be resolved for all monomers. The overall structure of AcrB and location of the gate loop is shown in **Fig 1A** and the asymmetric unit is depicted in **Fig 1B**. Data collection and refinement statistics appear in S1 Table.

Comparison of wild type AcrB and gate loop variants structures in space groups P2_1_ with previously reported structures, in either C2 or P1 space groups, shows that our structures are consistent with the asymmetric form of AcrB, and have high structural similarities as reflected by their low RMSDs (**S2 Table**). The structural asymmetry of AcrB trimer has been observed previously and has been shown to relate directly to its mechanism of substrate export [9, 10, 20]. Although our AcrB trimer structures in P2_1_ crystal forms are very similar to those in P1 and C2 crystal forms, the structures presented here exhibit distinct crystal contacts and crystal packing.

**Fig 2:**
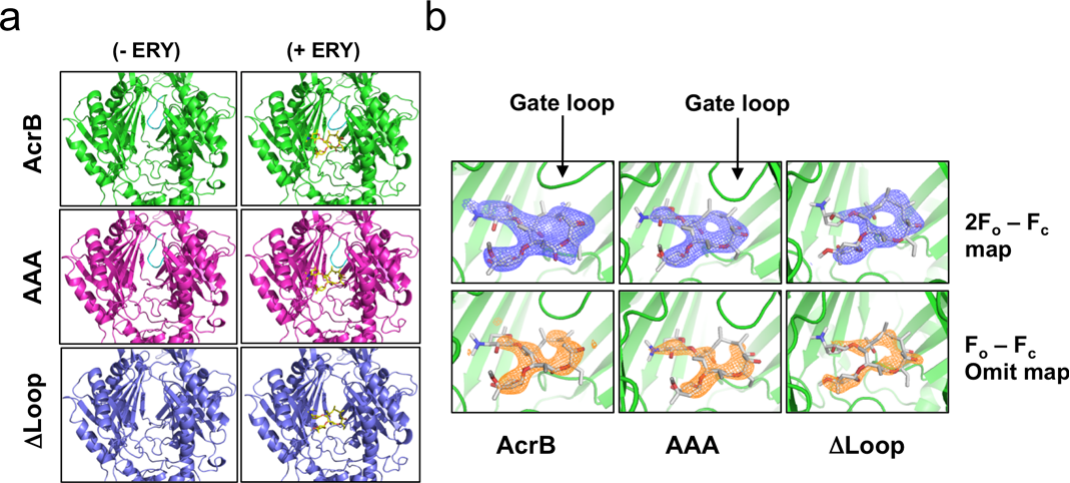
Erythromycin binding site and gate loop regions for wild type and variant AcrB. (**a**) Close-ups of the gate loop regions for reported crystal structures. (**b**) Erythromycin bound within the proximal pocket of AcrB and its variants. The electron density corresponding to the refined structure and the omit map are shown contoured at 1.0 **σ** (blue) and 2.5 **σ** (orange), respectively.

The gate loop regions of AcrB structures reported here are shown in **Fig 2A**. The wild type AcrB gate loop is clearly identifiable in the new P2_1_ crystal form and adopts a similar conformation to that observed in previous asymmetric AcrB crystal structures. Both gate loop variants also show well-resolved electron densities for the substituted residues. Overall the backbone atom positions for the AcrB(AAA) variant are similar to the wild type gate loop, while the AcrB(ΔLoop) structure reveals the gate loop deletion to be remarkably well tolerated. There are no obvious structural distortions elsewhere in the structure for either gate loop variant. These structures confirm that the gate loop substitutions do not perturb the overall fold of AcrB, its trimeric state, or its functional asymmetry.

### Gate loop involvement in the binding and export of erythromycin

The X-ray diffraction data obtained for AcrB crystals in the presence of erythromycin revealed electron density consistent with this antibiotic occupying the proximal pocket. The erythromycin molecule has a distinctive shape with a 14-membered ring and two sugar moieties that made its identification possible in the early stages of refinement. Electron density and omit maps for erythromycin are shown in **Fig 2.** The occupancy of erythromycin was estimated at 0.89 for the wild type structure, and 0.86 and 0.88 for the AcrB(AAA) and AcrB(ΔLoop) variants, respectively. Erythromycin-soaked crystals remain asymmetric, as in the apo-structures, and contain only 1 occupied binding site per trimer.

The erythromycin binding mode in the new P2_1_ crystal form is essentially identical to that previously observed for erythromycin-bound AcrB determined in space group C2 [10]. The one major difference between the gate loop variant structures and the wild type is the lack of a contact between erythromycin and the central phenylalanine residue of the gate loop (Phe617). In the wild type erythromycin-bound structure, this contact corresponds to ~10% of the erythromycin surface area. The absence of Phe617 in the gate loop variants does not lead to lower occupancy of erythromycin relative to wild type AcrB (**Fig 2B**).

No evidence of erythromycin bound to the distal pocket was seen for the wild type AcrB or either gate loop variant. The distal site has been defined largely on the basis of the structures of AcrB in complex with small substrates such as minocycline and doxorubicin [8, 9]. Evidence that such a site also exists for erythromycin comes from mutagenesis studies reporting amino acid substitutions in the distal site that deleteriously affect erythromycin export [11]. Simplistically, one might expect that removal of the gate loop would promote access and binding to the distal pocket. The results presented here suggest that disruption or even removal of the gate loop is not enough to facilitate occupation of the distal pocket by erythromycin—at least in crystallographic soak experiments involving isolated AcrB. Either the affinity for the distal binding site is very low or other factors and/or conformational changes are required to enable distal site binding. It is also plausible that residues from the gate loop, in particular F615, may also constitute part of the distal binding site necessary for erythromycin binding.

Both gate loop variants bound erythromycin; to investigate whether they supported erythromycin export we assessed erythromycin tolerance conferred by wild type and gate loop variant AcrB proteins. Minimum inhibitory concentrations (MICs) for erythromycin were measured in AcrAB-deficient *E*. *coli* strains complemented with plasmid-born AcrA and either wild type or gate loop variant AcrB. Equivalent expression levels for the wild type and variant AcrB were confirmed using Western blot analysis (**S1 Fig**). Strains expressing gate loop variants were both found to be highly susceptible to erythromycin, when compared to those expressing the wild type (MICs: wild type AcrB 500μM (or 367 μg/mL), AcrB(AAA) 125μM (92 μg/mL), AcrB(ΔLoop) 62.5μM (46 μg/mL), AcrB-negative control 31.25μM (23 μg/mL)). Consistent with previous findings [11, 12], these results suggest that the integrity of the gate loop is important for efficient erythromycin export by AcrB.

In summary, we have presented two AcrB variants in which the gate loop is disrupted, either by reducing bulky side chains or by removing it entirely. These variants express and fold similarly to the wild type AcrB, exhibit the same oligomeric state and trimer asymmetry, but are significantly impaired in their ability to confer erythromycin tolerance. Nonetheless, AcrB gate loop variants are still able to bind erythromycin. The work reconfirms the importance of the gate loop in the AcrB erythromycin-export mechanism while establishing that the interaction with the gate loop is not essential for the binding of erythromycin to the proximal binding pocket.

## Supporting Information

S1 Fig Western blot demonstrating uniform AcrB expression levels. Western blot analysis of cell extracts obtained from acrAB-deficient *E*. *coli* harbouring plasmids encoding wild-type, variant or no AcrB (control).
(PDF)

S1 Table Data collection and refinement statistics. (PDF)

S2 Table Structural superposition statistics. (PDF)
