# Supplementary figures and images for: Structures of Gate Loop Variants of the AcrB Drug Efflux Pump Bound by Erythromycin Substrate
Source: PLoS One. 2016 Jul 12;11(7):e0159154. doi: 10.1371/journal.pone.0159154 (PMC4942123; doi:10.1371/journal.pone.0159154)

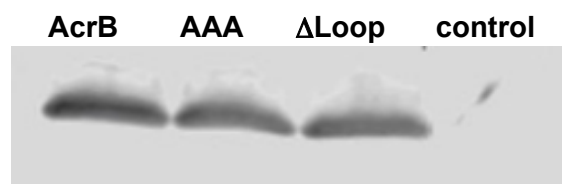

Supplement: S1 Fig — Western blot analysis of cell extracts obtained from acrAB-deficient E. coli harbouring plasmids encoding wild-type, variant or no AcrB (control). (PDF) [file pone.0159154.s001.pdf]
