# Supplementary material for: Structures of Gate Loop Variants of the AcrB Drug Efflux Pump Bound by Erythromycin Substrate
Source: PLoS One. 2016 Jul 12;11(7):e0159154. doi: 10.1371/journal.pone.0159154 (PMC4942123; doi:10.1371/journal.pone.0159154)
Supplement: S1 Table — (PDF) [file pone.0159154.s002.pdf]

S1 Table. Data collection and refinement statistics.

	AcrB	AcrB-ERY	AAA	AAA-ERY	Δloop	Δloop-ERY
**Data collection**						
Space group	P1 21 1	P1 21 1	P1 21 1	P1 21 1	P1 21 1	P1 21 1
Wavelength (Å)	1.0000	0.97949	0.97625	0.97949	0.97625	0.97625
Unit cell:						
a (Å)	151.84	150.95	152.28	151.18	152.07	150.06
b (Å)	156.86	155.67	157.49	156.77	157.78	154.58
c (Å)	218.58	217.01	219.16	218.17	219.39	215.74
α (°)	90.0	90.0	90.0	90.0	90.0	90.0
β (°)	92.7	92.7	92.7	93.1	93.1	92.4
γ (°)	90.0	90.0	90.0	90.0	90.0	90.0
Resolution range (Å)						
Overall (Å)	127.01-3.29	108.52-3.47	109.75-3.16	108.89–3.60	109.55-3.40	125.83-3.59
Outer shell (Å)	3.47 – 3.29	3.67 – 3.47	3.33 – 3.16	3.79 – 3.60	3.58 – 3.40	3.79 – 3.60
Completeness (%)	100.0 (100.0)	98.7 (96.1)	100.0 (100.0)	100.0 (100.0)	100.0 (100.0)	100.0 (100.0)
Unique reflections	153515	127313	176847	117756	142337	114348
Rpim (%)	7.2 (38.0)	7.1 (29.6)	5.7 (35.8)	9.2 (39.0)	5.6 (40.1)	7.6 (32.5)
Average redundancy	14.7 (14.0)	4.8 (4.1)	17.7 (17.9)	8.7 (8.9)	17.4 (17.1)	6.1 (5.8)
Average intensity, $< I/\sigma(I) >$	10.4 (2.1)	6.4 (2.0)	10.7 (2.2)	7.3 (2.5)	14.3 (2.4)	5.3 (2.0)
**Refinement**						
Resolution range (Å)	19.95 – 3.29	19.97 – 3.47	19.98 – 3.16	19.96 – 3.60	20.00 – 3.40	19.96 – 3.59
Rwork (%)	26.8	25.0	27.0	23.8	27.5	24.6
Rfree (%)	33.9	32.1	33.5	30.7	34.9	31.9
**Number of atoms**						
Protein	47560	47577	47523	47491	47319	47312
Ligands and metals	213	385	213	385	213	385
**B-factor ($Å^2$)**						
Protein	82.4	82.6	70.9	89.0	77.9	81.8
Ligands and metals	79.1	85.0	59.7	83.0	55.8	80.7
**Model Geometry Quality**						
RMSD bonds (Å)	0.013	0.012	0.013	0.012	0.013	0.012
RMSD angles (°)	1.77	1.78	1.78	1.73	1.87	1.78
**Ramachandran analysis**						
Fovoured (%)	91.2	91.4	89.0	91.4	90.6	87.9
Allowed (%)	6.9	6.5	9.0	6.5	6.8	9.1
Outliers (%)	1.9	2.1	2.0	2.1	2.6	3.0
