# Supplementary material for: Structures of Gate Loop Variants of the AcrB Drug Efflux Pump Bound by Erythromycin Substrate
Source: PLoS One. 2016 Jul 12;11(7):e0159154. doi: 10.1371/journal.pone.0159154 (PMC4942123; doi:10.1371/journal.pone.0159154)
Supplement: S2 Table — (PDF) [file pone.0159154.s003.pdf]

S2 Table. Structural comparison between monomers of AcrB, mutants, and reported structures in apo form (A) and in complex with erythromycin (B) forms (RMSD in Å).

(A) Apo structures

	Space group	A	B	C	D(A)[a]	E(B)[a]	F(C)[a]
2GIF	C2	0.70	0.61	0.65	0.95	0.71	0.77
2DHH	C2	1.20	1.03	1.15	1.41	1.06	1.24
2HRT	P1	0.68	0.68	0.74	0.96	0.72	0.81
AAA	P21	0.32	0.37	0.37	0.40	0.40	0.39
ΔLoop	P21	0.51	0.52	0.54	0.58	0.55	0.51

[a] between parenthesis are the monomers of 2GIF and 2DHH that consist of only 1 trimer

(B) Complex structures with ERY

	Space group	A	B	C	D(A)[a]	E(B)[a]	F(C)[a]
3AOC	C2	1.16	0.96	1.24	1.25	1.02	1.25
AAA	P21	0.38	0.33	0.34	0.40	0.36	0.37
ΔLoop	P21	0.49	0.47	0.41	0.50	0.45	0.47

[a] between parenthesis are the monomers of 3AOC that consists of only 1 trimer
